# Characterization of rabbit polyclonal antibody against camel recombinant nanobodies

**DOI:** 10.1515/biol-2022-0065

**Published:** 2022-06-15

**Authors:** Houssam-Eddin Khalaf, Hassan Al-Bouqaee, Manal Hwijeh, Abdul Qader Abbady

**Affiliations:** Division of Molecular Biomedicine, Department of Molecular Biology and Biotechnology, Atomic Energy Commission of Syria (AECS), P. O. Box 6091, Damascus, Syria

**Keywords:** nanobody, camel, polyclonal antibody, heavy-chain antibody, phage display

## Abstract

Nanobodies (Nbs) are recombinant single-domain fragments derived from camelids’ heavy-chain antibodies (HCAbs). Nanobodies are increasingly used in numerous biotechnological and medical applications because of their high stability, solubility, and yield. However, one major obstacle prohibiting Nb expansion is the affordability of specific detector antibodies for their final revelation. In this work, the production of a specific anti-Nb antibody as a general detector for camel antibodies, conventional cIgG, and HCAb, and their derived Nbs was sought. Thus, a T7 promoter plasmid was constructed and used to highly express six different Nbs that were used in a successful rabbit immunization. Affinity-purified rabbit anti-Nb rIgG was able to detect immobilized or antigen-bound Nbs via enzyme-linked immunosorbent assay, and its performance was comparable to that of a commercial anti-6× His antibody. Its capacities in dosing impure Nbs, detecting Nbs displayed on M13 phages, and revealing denatured Nbs in immune blotting were all proven. As expected, and because of shared epitopes, rabbit anti-Nb cross-reacted with cIgG, HCAbs, and 6× His-tagged proteins, and the percentage of each fraction within anti-Nb rIgG was determined. Anti-Nb is a promising tool for the checkpoints throughout the recombinant Nb technology.

## 1 Introduction

Antibodies and their recombinant derivative fragments are efficient tools in biotechnology. They are an important class of proteins that can be used for the prevention, treatment, and diagnosis of many diseases. Not surprisingly, immunoglobulins constitute the majority of the proteins in clinical trials [1]. Furthermore, because of their specificity and stability, antibodies are still among the most used markers for targeting drug nanocapsules to certain treated tissue in nanomedicine [2]. The development of antibody-based immunoassays, such as enzyme-linked immunosorbent assay (ELISA), has been proven to be very successful and important in research and clinical laboratories for detecting or quantifying various antigens [3]. Recently, molecular engineering of antibody fragments has emerged, and these recombinant proteins have started to break into several fields, including therapy, as a promising alternative to full-length antibodies [4].

A significant proportion of the functional antibodies in the bloodstream of the species of the Camelidae are devoid of light chains. These smaller bona fide immunoglobulins are referred to as heavy-chain antibodies (HCAbs) [5], and their antigen-binding fragment is comprised of a single domain (referred to as VHH or Nanobody^®^) with a molecular size of only ∼15 kDa. It is smaller compared to the single-chain variable fragments, also known as scFv (30 kDa), which are engineered by an artificial joining of both variable domains of heavy and light chains of conventional antibodies [6]. Nanobodies (Nbs) have many inherent advantageous properties besides their authenticate and unchanged original structure, including their low molecular mass, low immunogenicity [7], high affinity, high solubility, and stability [8]. Therefore, they can also target enzyme active sites that are not accessible to classical antibodies because of their huge size [9,10,11]. Nbs can act as crystallization chaperones, and this has been greatly appreciated in structural biology studies [12]. A sufficient supply of Nbs for research and applications is ensured through efficient recombinant production in micro-organisms, such as *Escherichia coli* [13], yeast [14,15], insect cells [16], and plants [17,18]. Also, Nbs can be easily expressed in living cells as specific *in vivo* “intrabodies” for tracing cytoplasmic and nuclear proteins [19,20]. Nbs are currently of high research interest for various pharmaceutical applications, including pathogens diagnosis [21,22], cancer therapy [15], autoimmune diseases [23], and neurodegenerative diseases [24]. More recently, they have been used in the specific targeting of drug nanocarriers [25,26] for cancer [27] and infectious diseases [28] therapy.

In the last decade, the interest in Nbs has increased in many laboratories around the globe, with many reports appearing each year about isolating interesting Nbs against a broad spectrum of antigens, including haptens [29], viruses [30,17,26], toxins [31,32], and even pathogens [33], for example, Brucella [34,35,36], Trypanosoma [37,28], and Taenia solium [38]. Their immanent success was proven in many fields, and they have been used as research tools in several laboratories and for a variety of applications [39,40].

Nbs are routinely isolated after multiple steps, starting with the immunization of a camel with the antigen, constructing a cDNA library containing the VHH genetic repertoire from the camel B cells pool, and finally screening the library by phage display against the immobilized antigen [41,42]. Hence, assuring a good immune response is crucial before pursuing the procedure and constructing a laborious and expensive Nb “immune” library. Camel antibody testing as a bulk for their ability to recognize the antigen in ELISA is sometimes not sufficient to assess the immune response, and additional steps must be taken to ensure that HCAbs are involved as well in that response. Because Nbs are derived from the variable domain of HCAbs, such information is indispensable. Practically, three distinct IgG fractions (IgG1, 2, and 3) with different molecular weights can be separated from camel serum by differential adsorption on protein-A and protein-G columns [36]. While the so-called IgG1 subclass represents conventional antibodies, IgG2 and IgG3 subclasses contain HCAbs [5]. The percentage of these different subclasses shows different values in the sera of camelids; it might reach 50–80% in camels, whereas it is about 10–25% in South American llamas [43]. HCAb subclasses are important in camelid immunity, especially in response to antigens from pathogenic origins [44,45,5].

A limiting factor for Nb applications as well as for investigations into camel immunity has been the scarcity of affordable and specific detecting reagents. For example, antigen-bound Nbs are usually revealed using recombinant tags, added at one of their ends like 6× His and/or hemagglutinin (HA) [46,42], c-Myc [47], or FLAG [48]. Most of these tags or domains have commercialized antibodies that certainly differ in the optimal working conditions and concentrations.

Recently, we have reported a modest attempt to prepare and characterize a general antibody as a detecting reagent for camel IgGs and their recombinant Nbs based on a commercially available polyclonal antibody directed against camel serum [49]. Here, we produced a specific and reactive rabbit polyclonal antibody against a cluster of different Nbs. Thus, it was named anti-Nb rIgG. Since Nbs are structurally derived from camel IgGs, particularly HCAbs, the anti-Nb rIgG was useful to elucidate the common epitopes between the Nb, HCAb, and conventional cIgG. A significant fraction of anti-Nb rIgG recognized the 6× His tag as a distinct domain of the recombinant Nb structure. Such a secondary anti-Nb antibody, besides its usefulness in the detection and titration of Nbs by various immune methods, is itself a useful source to prepare anti-HCAbs, anti-cIgG, and anti-His IgGs, which are essential antibodies during Nb preparation technology.

## 2 Materials and methods

### 2.1 Antigens and antibodies

For ELISA, the detection of antigen-bound Nbs was mostly accomplished using a rabbit anti-6× His antibody (Bethyl Laboratories Inc., 1:2,000 v-v dilution). Polyclonal antisera antibodies (used at 1:1,000) from goat against mouse, rabbit (1:3,000), and human (IgG, IgM, IgA, IgE, and total), or rabbit against the horse, camel (1:5,000), sheep (Koma Biotech Inc.), goat, chicken (Invitrogen, 1:3,000), and bovine were from Bethyl Laboratories Inc. (unless indicated) and were (except the anti-camel) conjugated to the horseradish peroxidase (HRP). For Nb preparation, pMES4 phagemid, pHEN6, and *E. coli* strains (TG1 and WK6) were kindly provided by Prof. Serge Muyldermans (VUB, Brussels, Belgium). As controls, plasmid construct pT7-His expressing a fusion protein of the superfolder green fluorescent protein (*sf*GFP), and the tobacco etch virus (TEV) protease with a C-terminal 6× His tag (53.7 kDa) [50] and a pRSET-TEV-GH plasmid expressing human growth hormone (hGH) with N-terminal 6× His tag (27 kDa) [42,51], were prepared as previously described. *E. coli* BL21(DE3)-Gold expression of these recombinant proteins followed by metal affinity purification was performed using the standard protocol. As an antigen for Nbs, commercial untagged rhGH was obtained from Sigma, and its polyclonal antibody (R-anti-GH, 1:3,000, homemade) was prepared in a previous study [51]. Another antigen for Nbs, GFPuv was expressed using pGV4940-GFPuv plasmid (kindly provided by Prof. Serge Muyldermans) in WK6, and its control detection was carried out using a rabbit polyclonal antibody (R-anti-GFP, 1:3,000, homemade) [52]. Syrian isolate of the broad bean mottle virus (BBMV, 0.4 mg mL^−1^) and its polyclonal rabbit antibody (R-anti-BBMV, 1:3,000, homemade) were kindly provided by Dr. Safa Kumari (Plant Virology lab., International Center for Agricultural Research in the Dry Areas (ICARDA), Syria) and used as an antigen for Nbs [17].

### 2.2 Expression and purification of soluble Nbs

A plasmid for Nb overexpression under the control of a powerful T_7_ promoter was constructed using the pRSET-a plasmid (Invitrogen) backbone. For this aim, firstly, a long DNA fragment (1,401 bp) was extracted from the pMES4 plasmid by partial digestion with *Nde*I/*Eco*RI and then sub-cloned into pRSET-a plasmid resulting in a new plasmid construct named pRMES4. Secondly, a very short DNA fragment (80 bp) was sub-cloned from the pHEN6 plasmid into pRMES4 using *Nco*I/*Eco*RI resulting in a new plasmid pRMES6, which was confirmed by sequencing. Six different Nbs were used in this study: NbGH01 (Accession No. KJ732842) and NbGH04 (Accession No. KJ732845) against hGH [42], NbGFP03 (Accession No. KJ732837) and NbGFP08 (Accession No. KJ732841) against *sf*GFP [46], and NbBBMV01 (Accession No. MF001020) and NbBBMV10 (Accession No. MF001026) against BBMV [17]. Their encoding sequences were extracted from their respective pMES4 derivative plasmids after *Pst*I/*Bst*EII digestion before being sub-cloned in pRMES6.

Confirmed plasmid constructs were used to transform *E. coli* BL21 (DE3)-Gold (Agilent Technologies) by electroporation. Large-scale production was performed in 250 mL shake flasks by growing the bacteria in Luria–Bertani (LB) broth medium (1% tryptone, 0.5% yeast extracts, and 1% NaCl) supplemented with 100 µg mL^−1^ ampicillin until an optical density (at 600 nm) of 0.5 was reached, and then, expression was induced with 0.5 mM isopropyl β-d-1-thiogalactopyranoside (IPTG; Sigma) for 16 h at 28°C. In the case of Nbs in pMES4-derived plasmids, expression of Nbs was performed in *E. coli* WK6 using standard procedure [46]. The next day, cells were pelleted, and the periplasmic proteins containing Nbs were extracted by osmotic shock. Using FPLC, this periplasmic extract was loaded on a 5 mL nickel charged HisTrap HP column (GE Life Sciences), and after washing, the bound proteins were eluted with a 500 mM imidazole buffer using a previously published standard procedure [53]. Size exclusion on *Superdex 200*-10/30 gel filtration column (GE Life Sciences) was carried out as a final purification step for Nbs. The column was equilibrated with 60–90 mL of sodium phosphate buffer at a 1 mL min^−1^ flow rate before injecting 0.5 mL of the Nb sample (∼4 mg). The eluted fraction was concentrated on Vivaspin concentrators with a 5–10 kDa molecular mass cutoff. Purified Nbs were separated by 15% SDS-PAGE and then visualized by Coomassie blue staining. The absorbance at 280 nm and the extinction coefficient, as calculated from the amino acid sequence of each Nb, were used to determine the concentrations of the purified Nbs, which were finally adjusted to 1 mg mL^−1^ and stored at −20°C. Equal volumes of all six different Nbs were used to prepare the Nb mixture (1 mg mL^−1^, ∼0.16 mg mL^−1^ each) for use in the following steps.

### 2.3 Rabbit and chicken immunization

For immunization, three adult white female rabbits aged 2 months (weighing ∼2 kg) and three white Leghorn laying-eggs chickens aged 8 months were used in this study. Nb mixture (0.5 mg/injection/animal) in 1 mL PBS was mixed with an equal volume of Freund’s complete adjuvant (Bio Basic Inc.) to form a stable emulsion used for the first immunization at 2–4 different sites. Subsequently, three booster injections were given mixed with incomplete Freund’s adjuvant at 15-day intervals. Blood samples were collected from the chicken wing vein and marginal ear vein of rabbits. This was achieved before immunization (day 0) and at regular intervals before each injection. Final bleeding (30 mL from rabbits) was done 15 days after the last boost on day 56. One rabbit was left unimmunized, and its serum was used as a negative control for affinity purification experiments. After the experiments were over, all these animals were euthanized or put down.


**Ethical approval:** The research related to animal use has been complied with all the relevant national regulations and institutional policies for the care and use of animals and has been validated and approved by an Institutional Reviewing Board and Ethical Committee of the Atomic Energy Commission of Syria (AECS). The committee’s reference number for the project is 200/2016.

### 2.4 Purification of chicken IgY from egg yolk

Eggs from immunized chickens (around day 56) were collected. The method of purifying IgY from egg yolk involves carefully removing the egg white and yolk membrane from the broken egg and then eliminating lipids by precipitation in 3.5% polyethylene glycol 6000 (PEG_6000_, Carl Roth) and filtration, followed by the precipitation of total IgY from the supernatant of the previous step using 12% PEG_6000_. Finally, IgY pellet was washed several times before dissolving in 2 mL PBS [54]. After dialyzing against PBS, IgY concentration (mg mL^−1^) was measured photometrically at 280 nm and calculated with an extinction coefficient of 1.33 before being stored (5 mg mL^−1^) at −20°C. The purity of the extracted IgY was around 80% with >50 mg total IgY recovery per egg.

### 2.5 Purification of rabbit anti-Nb polyclonal antibody

Polyclonal rabbit anti-Nb antibody (rIgG) was purified from 5 mL rabbit serum (day 56) by affinity chromatography on a 5 mL HiTrap Protein A column (GE Life Sciences) according to the manufacturer’s instructions. Briefly, binding of rIgG in the serum to the column was performed in 0.02 M sodium phosphate, pH 7.0. Next, the column was washed extensively before rIgG was eluted with 0.1 M citric acid, pH 3.0. Eluted rIgG was collected and immediately neutralized to physiological pH with 1 M Tris-base buffer, pH 9.0, and then dialyzed against PBS before concentrating to 1 mg mL^−1^ on Vivaspin concentrators with a molecular mass cutoff of 50 kDa (Vivascience).

### 2.6 Purification of camel IgG subclasses

Different IgG subclasses (IgG1, 2, and 3) were separated from 5 mL of camel serum as previously described [49] by differential adsorption on Hitrap-Protein A and Hitrap-Protein G columns (GE Life Sciences). Camel total antibodies (cIgGt) were purified from the same diluted serum by direct affinity chromatography on a Protein A column in a manner similar to the purification of rabbit IgG. Once eluted, all IgG fractions were neutralized with 1 M Tris pH 9.0, dialyzed against PBS, quantified, diluted to 1 mg mL^−1^, and stored at −20°C.

### 2.7 Affinity purification of specific rIgG fractions

Four *N*-hydroxysuccinimide (NHS)-activated Sepharose 1 mL columns (GE Life Sciences) were used for affinity purification of specific anti-Nb rIgGs from total rabbit antibodies. Concentrated ligands (Nb mixture, cIgG1, cIgG2 and 3 mixture, and *sf*GFP-TEV as a C-terminal 6× His tagged protein) were prepared (20 mg) in coupling buffer (0.2 M NaHCO_3_, 0.5 M NaCl, pH 8.3) and applied into NHS-activated column to allow conjugation to take place for 30 min at 20°C. Any excess of active NHS groups that have not been coupled was deactivated by sequential washing with quenching buffer (0.5 M ethanolamine, 0.5 M NaCl, pH 8.3) and then with washing buffer (0.05 M sodium phosphate, 0.15 M NaCl, pH 7.0). Total rabbit IgGs (40 mg) were passed through the conjugated columns to capture ligand-specific rIgG by affinity. Unbound rIgG (flow through), which was empty of specific antibodies, was washed away with washing buffer before eluting specific antibodies with 3–5 column volumes of 0.1 M citric acid buffer, and then, they were neutralized with 1 M Tris-HCl buffer, pH 9.0. Affinity-purified rIgG samples were subjected to buffer exchange against 0.05 M sodium phosphate buffer pH 7.0 and, then, concentrated down to 1 mg mL^−1^.

### 2.8 ELISA

Several ELISA formats were employed in this study using Maxisorb 96-well plates (Nunc) which were coated overnight at 4°C with antigens (Nb mixture, camel IgG subclasses, hGH, GFP, BBMV, N- or C-terminal 6× His tagged proteins) as indicated for each experiment (1 µg mL^−1^ and 100 µL per well). After coating, ELISA plates were washed three times with washing buffer TBS-T (20 mM Tris-base, 150 mM NaCl, 0.05% tween-20, pH 7.5). Residual protein binding sites in the wells were blocked for 1 h at RT with 5× blocking buffer (5% skim milk in TBS-T). For direct ELISA, different antisera HRP-conjugated antibodies were diluted in 1× blocking buffer and added to the wells for 1 h at RT, while indirect ELISA was used for analyzing sera from immunized animals and detecting immobilized or antigen-bound Nbs. After the removal of the blocking buffer, immunized rabbits sera (1:2,000), rabbit anti-camel (1:5,000), chicken sera (1:100), chicken IgY (10 µg mL^−1^), serial dilutions of rabbit anti-Nb rIgG, rabbit anti-GH, rabbit anti-GFP, rabbit anti-BBMV or six different pure Nbs (1:500) were all diluted in 1× blocking buffer and added to the wells for 1 h at RT. After 3 washes, the detection of antigen-bound Nbs was performed with rabbit anti-Nb rIgG (1:500) or commercial rabbit anti-6× His (1:2,000) antibodies. After plate washing, all rabbit antibodies were detected by 1 h incubation at RT with goat anti-rabbit antibody conjugated to HRP at 1:3,000 in 1× blocking buffer. After an additional five washes, bound conjugates (from direct and indirect ELISA) were detected with 3,3′,5,5′-tetramethylbenzidine (TMB) substrate (Sigma), and then, the reaction was stopped after 15 min with the addition of 1 M H_2_SO_4_. The spectroscopic absorbance of the enzymatic reaction was measured in an automated plate reader Multiskan™ FC (Thermo) at a wavelength of 450 nm.

### 2.9 Quantification of Nbs by competitive ELISA

The EC_50_, defined as the molar concentration giving the half-maximal OD, was determined by titrations of rabbit anti-Nb rIgG (∼1:3,000) or rabbit anti-6× His (∼1:5,000) on an adsorbed Nb mixture (1 µg mL^−1^) using the already mentioned indirect ELISA. For the purpose of Nb titrations by competitive ELISA, an Nb mixture (1 µg mL^−1^) was prepared in carbonate buffer and used to coat (100 μL) in a 96-well ELISA plate at 4°C overnight. After blocking and washing (as in a standard ELISA), the range of Nbs concentrations (0.1–100,000 ng mL^−1^) was prepared (100 μL) and first incubated for 1 h at RT with either anti-Nb rIgG (1:5,000) or with anti-6× His (1:5,000) and then transferred directly to the wells containing the immobilized Nb mixture and incubated for an additional 1 h at RT. Subsequent steps were performed as already mentioned in standard ELISA.

### 2.10 Phage ELISA

Propagation of M13K07 helper phages (GE Life Sciences) after infection of *E. coli* TG1 cells was performed using a standard procedure [55]. Similarly, TG1 cells were transformed with pMES4 phagemid containing the coding sequence of NbGFP08 and grown before being infected with helper M13 phages to rescue the recombinant virions (M13-Nb) displaying Nbs on their tips [55]. Precipitation of M13 phages from the supernatant was done using 5:1 v:v volume of PEG_6000_/NaCl (20% PEG_6000_ and 2.5 M NaCl). Finally, phages were pelleted down by centrifugation and resuspended in 0.5 mL of PBS containing 7% dimethyl sulfoxide (Sigma). Phage concentration was measured using a spectrophotometer at OD_260_ (1 OD = 10^11^ pfu mL^−1^) and adjusted to 10^10^ pfu mL^−1^. Phage titration ELISA was achieved using immobilized either rabbit anti-M13 (1:3,000, homemade) [55] or anti-Nb rIgG (1:500) for capturing, and a mouse anti-M13 antibody conjugated to HRP (1:3,000, GE Life Sciences) for detecting phage particles, and the remaining steps were performed in a manner similar to standard ELISA.

### 2.11 WB and Coomassie blue staining of SDS-PAGE

SDS-PAGE was performed using the Bio-Rad mini-Protean II system following the manufacturer’s instructions. Gels were prepared using stacking gel 5% and running gel 15%. After electrophoresis, the gel was stained with Coomassie blue for 2 h followed by destaining in 5% acetic acid and 10% methanol. For immunoblotting, the separation was carried out to 0.25 µg/lane of the different antigens (cIgG1, cIgG2, cIgG3, C- and N-terminal tagged proteins, and Nbs separately or in the mixture), which were then blotted on 0.45 µm nitrocellulose membranes (Bio-Rad) using 1× blotting buffer (25 mM Tris-base, 200 mM glycine, 0.1% SDS and 20% methanol). After adding blocking buffer (TBS-T with 5% skim milk), the indicated dilutions of the primary antibodies were added to the membranes for further incubation for 1 h at RT. After several washes with TBS-T, blots were finally incubated with polyclonal anti-rabbit HRP-conjugated antibody (1:3,000) for 1 h at RT. Bands were revealed by adding the chromogen substrate AEC (3-amino-9-ethylcarbazole) prepared in acetate buffer containing H_2_O_2_.

## 3 Results

### 3.1 Expression and purification of soluble Nbs

**Figure 1:**
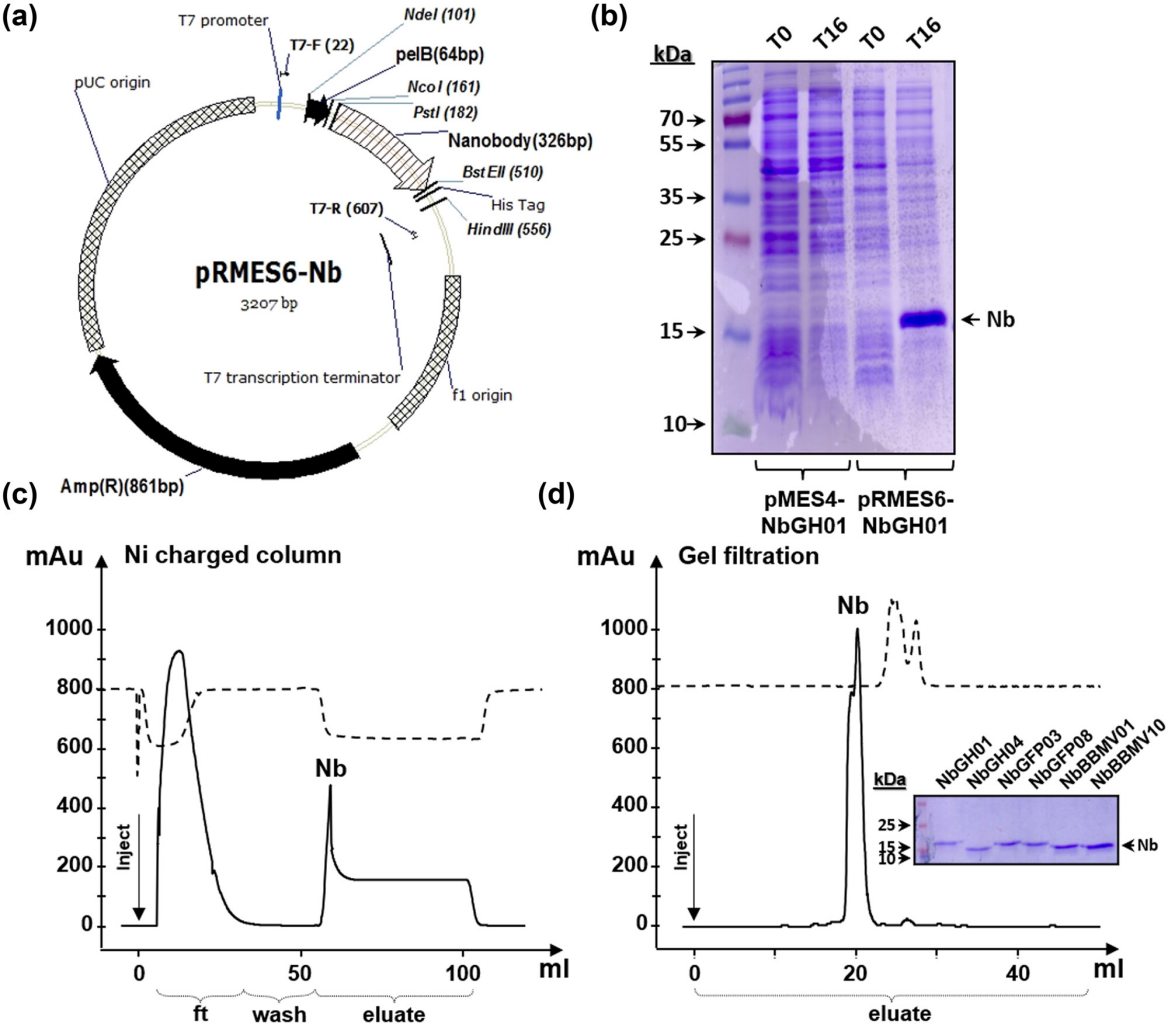
Expression and purification of Nbs. (a) Map of the pRMES6-Nb plasmid construct showing the inserted Nb coding sequence. The most important elements of the plasmid are indicated, these include T_7_ promoter, 6× His tag downstream the two restriction sites (*Pst*I and *Bst*EII) used for Nb-coding sequence insertion. The pelB leader signal at the N-terminal side of Nb is shown. (b) SDS-PAGE (acrylamide 15%) of protein samples before (lanes 1 and 3) and after (lanes 2 and 4) 16 h of IPTG induction using pMES4 and pRMES6 plasmids expressing NbGH01. (c) Diagram of Nb purification (case of NbGH01) using Ni^+^-charged column installed on FPLC AKTAprime system. The continuous line represents the absorbance of the eluate, and peaks of the flow-through (ft) and purified Nb are indicated. The dashed line represents the conductivity of the eluate. (d) Purified Nb (case of NbGH01) obtained after affinity chromatography was injected in *Superdex 200* 10/300 GL column at flow-rate of 1 mL min^−1^, and a peak of Nb fraction was collected after 18 mL of retention. (d, inset) SDS-PAGE (acrylamide 15%) of the six different Nbs (∼2 µg/lane) used in this study after the same steps of purification. Coomassie blue staining was used for bands visualization.

The coding sequences of Nbs are usually under the control of a weak P*lac* promoter in plasmids, such as pHEN4, pHEN6, or pMES4. We have started by constructing a pRMES6 plasmid expressing Nbs in high yields under the control of the pT_7_ promoter, using a pRSET-a backbone and an adapter fragment from the plasmid pHEN6 (Figure 1a). This pRMES6 plasmid construct, similar to pHEN6, can receive any Nb coding sequence simply by sub-cloning with *Pst*I/*Bst*EII from the original plasmids (pHEN4 or pMES4) used in the Nb technology. This is essential as these two restriction enzymes are frequently used in the construction of Nb libraries, and thus, their occurrence within the coding sequences of newly isolated Nbs should be rare. The expressed Nb from pRMES6 conserves all original features; the PelB signal for periplasmic localization as well as the C-terminal 6× His tag. Remarkably, replacing pMES4 with pRMES6 resulted in enhancing the expression of multiple Nbs, like NbGH01, as shown by SDS-PAGE separation and Coomassie blue staining (Figure 1). Purification of produced Nbs was possible due to their 6× His tag by chromatography using a nickel-charged column (Figure 1c). Moreover, pure Nbs were further purified using size exclusion chromatography, resulting in a single peak after 20 mL retention (Figure 1d). A standard procedure was applied to express and purify five other Nbs recognizing three different antigens: NbGH04 recognizing hGH, NbGFP03&08 recognizing GFP, and NbBBMV01&10 recognizing the plant broad bean mosaic virus (BBMV). The expression yields of these soluble Nbs using the new expression system were estimated between 15 and 125 mg L^−1^ of bacterial culture. A mixture of these six Nbs was prepared and used in this study.

### 3.2 Cross-reactivity assay of antisera against camel IgGs and Nb mixture

**Figure 2:**
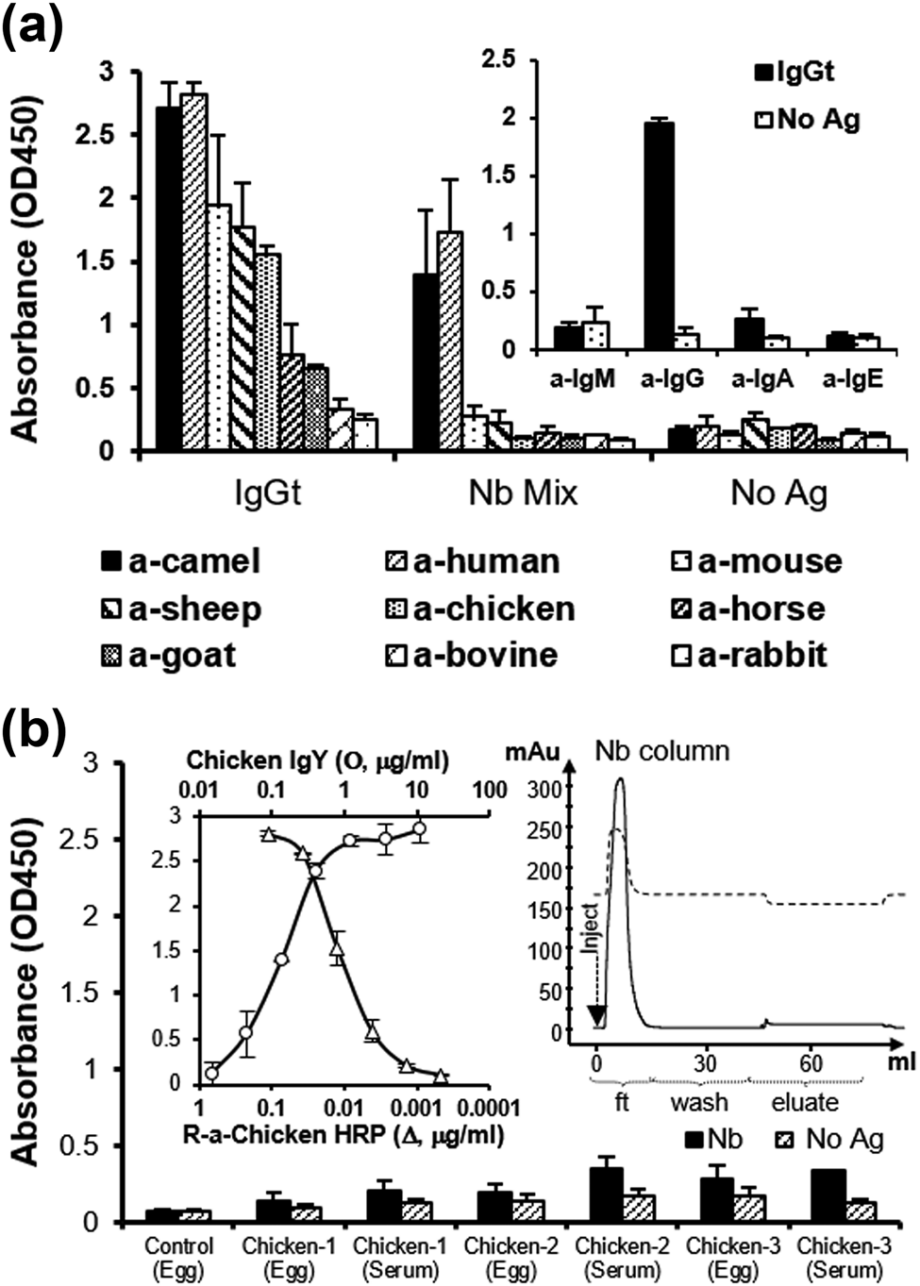
Evaluation of antisera cross reaction with camel IgG and Nb mixture. (a) The reactivity of several antisera with immobilized camel total IgG (IgGt) and Nb mixture was tested by direct ELISA. (a, inset) Different anti-human immunoglobulin classes (M, G, A, and E) were tested by direct ELISA against immobilized camel IgGt. (b) The immune response of three immunized chicken with Nb mixture was evaluated by testing the reactivity of their IgG in the serum (1/100) and IgY extracted from the egg yolk (10 µg mL^−1^) against immobilized Nbs (1 µg mL^−1^) by indirect ELISA. Detection of chicken Ig was done using a rabbit anti-chicken antibody conjugated to HRP. (b, left inset) Assessment of the reactivity (Δ) of serial dilutions (0.1–0.003 µg mL^−1^) of the rabbit anti-chicken-HRP antibody toward purified IgY (1 µg mL^−1^) and the sensitivity (O) of the anti-chicken-HRP (0.3 µg mL^−1^, ∼1:3,000) in the presence of different concentrations of the immobilized IgY (0.01–10 µg mL^−1^). (B, right inset) 5 mL of purified IgY (5 mg mL^−1^) was injected on the Nb-affinity column and, after 50 mL of retention, elution was performed.

The reactivity of several available antisera was tested by ELISA against total cIgG, purified from camel serum on a protein-A column and Nb mixture (Figure 2a). As expected, the anti-camel antibody was able to detect cIgGs and Nbs. Interestingly, the anti-human antibody was able to detect cIgGs and Nbs similarly to the anti-camel antibody, while other animal antisera showed variable sensitivity toward cIgGs without being able to recognize Nbs (Figure 2a) significantly. To confirm cross-reactivity of the anti-human serum with cIgGs, antibodies against the four major classes of human immunoglobulins (IgM, IgG, IgA, and IgE) were tested in the presence of immobilized cIgG, and it appears that anti-human IgG is solely responsible for this cross-reaction (Figure 2a, inset).

### 3.3 Assessing chicken as a potential host for immunization with Nbs

As inferred from the previous test, anti-chicken serum showed relatively high reactivity toward camel IgG. However, no cross-reactivity against Nbs could be observed. Based on this observation, three chickens were subcutaneously immunized with five doses of Nb mixture at an interval of 2 weeks. Sixty-five days after the start of chicken immunization, IgY was prepared from their egg yolk and used together with a serum sample to test the immune response against Nbs by ELISA (Figure 2b). The results were disappointing since no significant reactivity could be observed in the samples from the three birds compared to those from the control one. This low ELISA signal is in no way associated with a problem in the interaction between chicken IgY and the used commercial anti-chicken-HRP antibody since a very high dilution of this conjugated antiserum (∼0.1 μg mL^−1^) is sufficient to detect very low quantities of immobilized IgY (>0.03 μg mL^−1^) by ELISA (Figure 2b, left inset). Also, applying IgY from immunized birds into the affinity column conjugated with an Nb mixture did not result in a distinguishable pure fraction peak, indicating the absence of significant immune response against Nbs (Figure 2b, right inset).

### 3.4 Rabbit immunization with Nbs and the purification of anti-Nb rIgG

**Figure 3:**
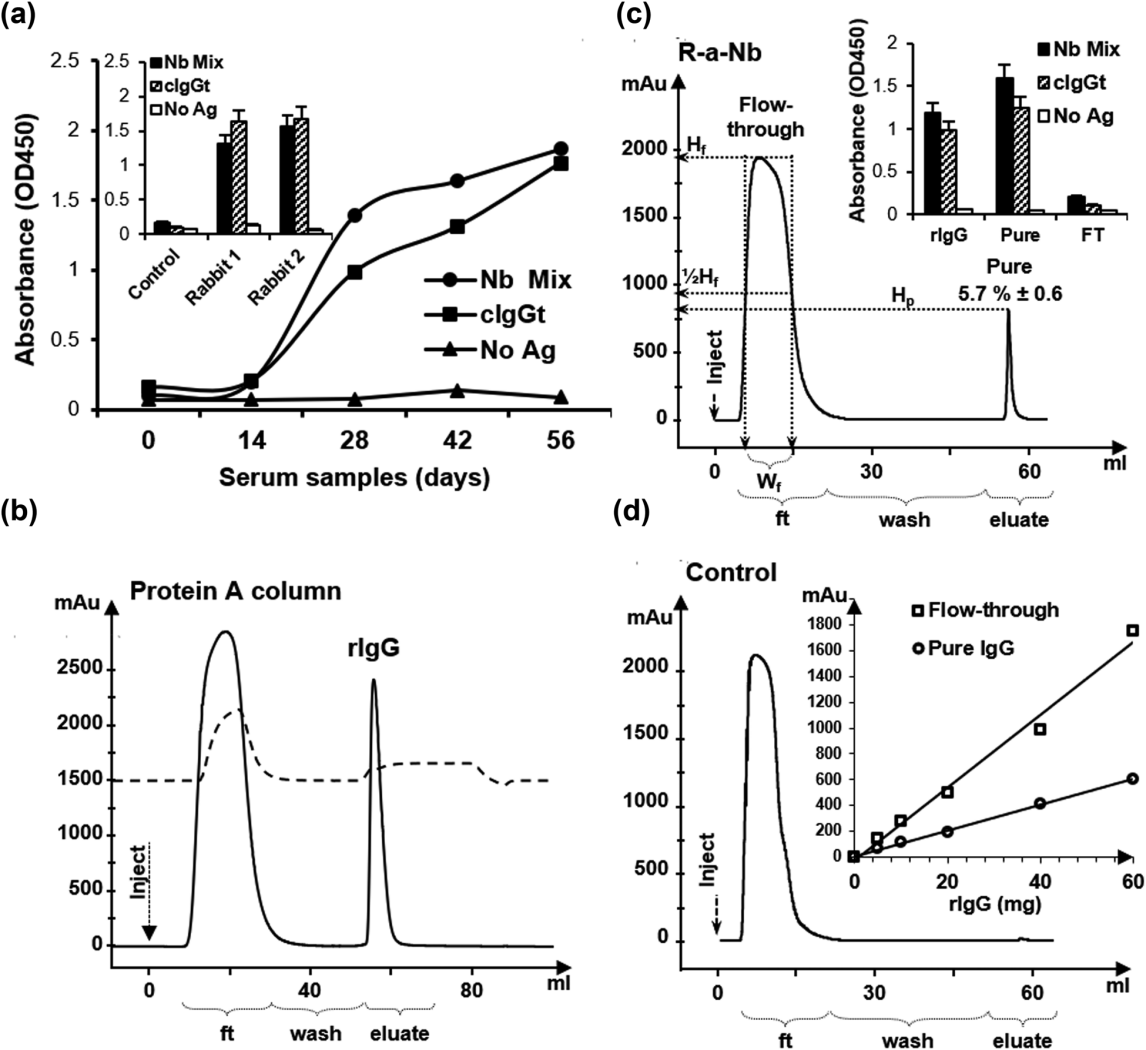
Evaluation of the immune response raised in rabbits against the Nb mixture. (a) The reactivity of rabbit sera (diluted to 1:2,000) taken at different time points (days) during immunization was tested by ELISA in the absence (No Ag) or the presence of immobilized Nb mixture (1 µg mL^−1^, Nb Mix). (a, inset) Indirect ELISA of the reactivity of the final point (day 56) of rabbit sera (diluted to 1:2,000) from three rabbits (two immunized and one control) against camel IgG and Nb mixture (1 µg mL^−1^, Nb Mix). (b) Purification of IgG from rabbit serum by affinity chromatography. Five milliliters of diluted (1:1 in PBS) rabbit serum (day 56) was injected onto a HiTrap protein-A column and washed with phosphate buffer (wash) to remove unbound proteins flow through (ft), before eluting pure rabbit rIgG (eluate). Five milliliters of pure rIgG (10 mg mL^−1^) from the immunized (c) and control (d) rabbits was injected on the Nb-affinity column and after 50 mL of retention, an elution was performed to recover pure anti-Nb fraction. (c, inset) Original rIgG as well as pure and ft fractions were tested in ELISA against immobilized cIgG and Nb mixture (1 µg mL^−1^). (d, inset) The binding capacity of the Nb-affinity column was tested by passing different amounts of rIgG (0–60 mg) for purification. Absorbance (mAu) of the ft and pure anti-Nb rIgG peaks after each injection were presented.

Two adult female rabbits were immunized with four doses of Nb mixture by subcutaneous injections. Blood samples were collected at several time points from the start of immunization and tested in ELISA against an immobilized Nb mixture, demonstrating the rise of a specific immune response after 15 days of immunization onward (Figure 3a). Rabbit immune response against Nbs increased almost exponentially up to day 28, after which it maintained the same level until the final bleeding at day 56. Indirect ELISA showed a similar level of reactivity of the final point sera (day 56) from two different immunized rabbits against camel IgG and Nb mixture compared to control unimmunized animal (Figure 3a, inset). Rabbit total IgGs (rIgG) were prepared from the serum at day 56 and purified on the protein-A column (Figure 3b). The percentage of anti-Nb fraction in rIgG was determined by achieving the second step of affinity purification on an Nb-conjugated column (Figure 3c). This resulted in a pure fraction of anti-Nb rIgG of about 5.7 ± 0.6% of total rIgG that showed higher reactivity in ELISA against immobilized cIgG and Nb mixture than total rIgG (Figure 3c, inset). Interestingly, such a fraction was absent when the same purification procedure was applied using rIgG from unimmunized animals (Figure 3d). Calculating the percent of pure anti-Nb from total rIgG was done using the equation: (*W*_p_ × *H*_p_) × 100/(*W*_f_ × *H*_f_), where *H*_p_ and *H*_f_ are the heights (mAu) of the pure and flow-through peaks, *W*_p_ and *W*_f_ are their widths at *H* ^1/2^, respectively (Figure 3c). The capacity limit of the Nb-conjugated column and the accuracy of the last equation were tested using several amounts of rIgG for purification, and a linear correlation was found between the peak values of pure and flow-through fractions and the amounts of injected rIgG used in the purification (Figure 3d, inset).

### 3.5 Characterization of anti-Nb rIgG

**Figure 4:**
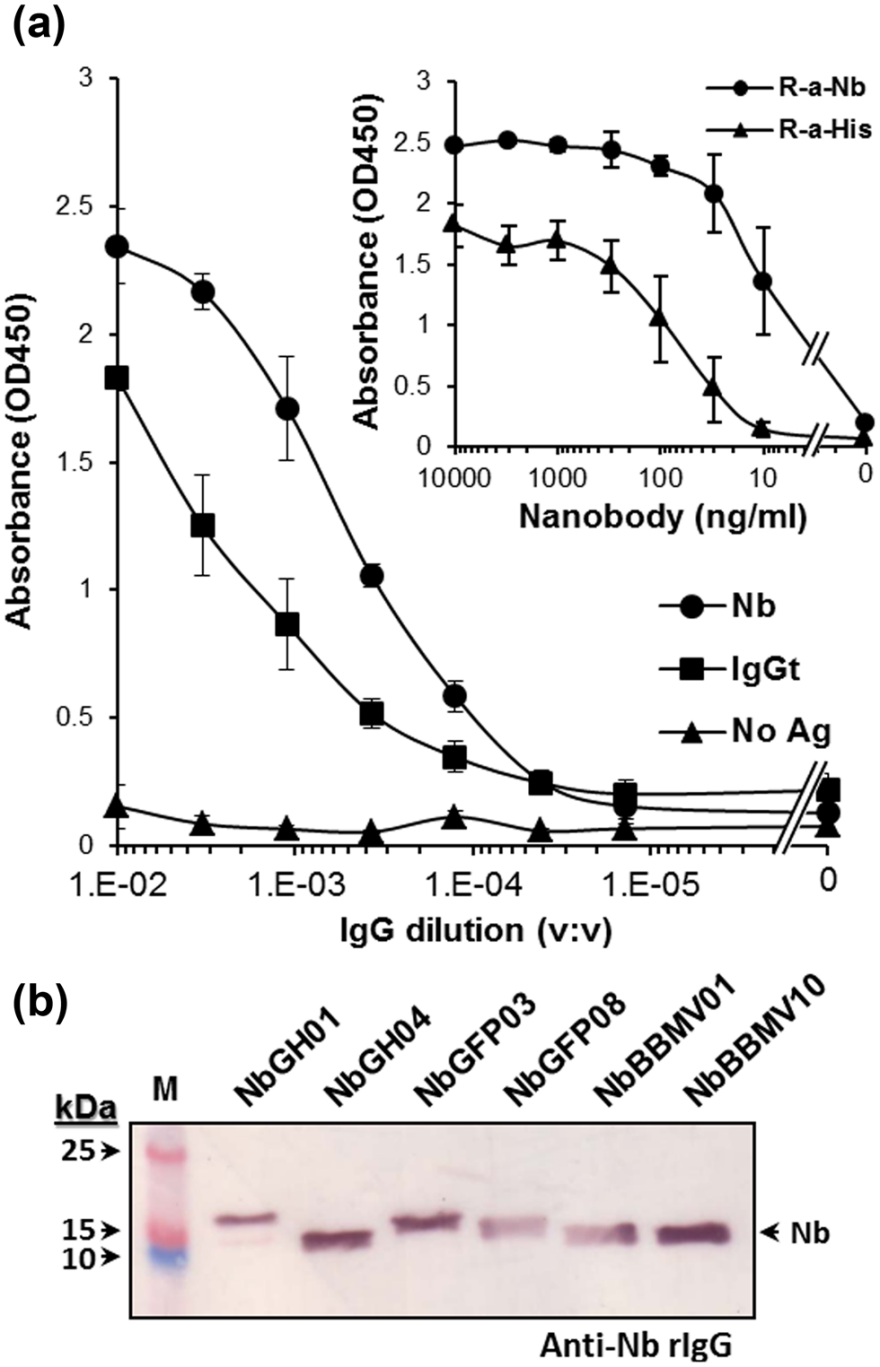
Characterization of anti-Nb rIgG. (a) Purified rabbit anti-Nb (1 mg mL^−1^) was tittered by ELISA using the indicated serial dilutions (v:v) in the absence (No Nb) or the presence of Nb mixture (Nb, 1 µg mL^−1^) or total camel IgG (IgGt, 1 µg mL^−1^). (a, inset) The sensitivity of anti-Nb rIgG (1:500) and a rabbit anti-6× His tag antibody against serial dilutions of immobilized Nbs (ng mL^−1^) was shown by ELISA. (b) Immunoblotting of several pure Nbs (0.2 μg/lane) using anti-Nb rIgG (1:500). The location of Nb bands is indicated and defined as ∼15 kDa by comparing it to the protein molecular weight ladder in the first lane (M).

Titration of purified anti-Nb rIgG by ELISA showed high reactivity toward an immobilized Nb mixture and, to a lesser extent, to the total camel IgG (cIgGt) and important median effective concentration (EC^50^) toward its antigen, in the range of 0.3 µg mL^−1^ (1:3,000 dilution v-v) (Figure 4a). Another ELISA confirmed that anti-Nb rIgG is more efficient than anti-6× His antibody in detecting low concentrations (∼10 ng mL^−1^) of immobilized Nbs (Figure 4a, inset). Moreover, anti-Nb rIgG was able to similarly detect, by immunoblotting, all different Nbs in the mixture used to immunize rabbits (Figure 4b).

### 3.6 Application of anti-Nb rIgG in immunodetection of Nb-specific antigens

**Figure 5:**
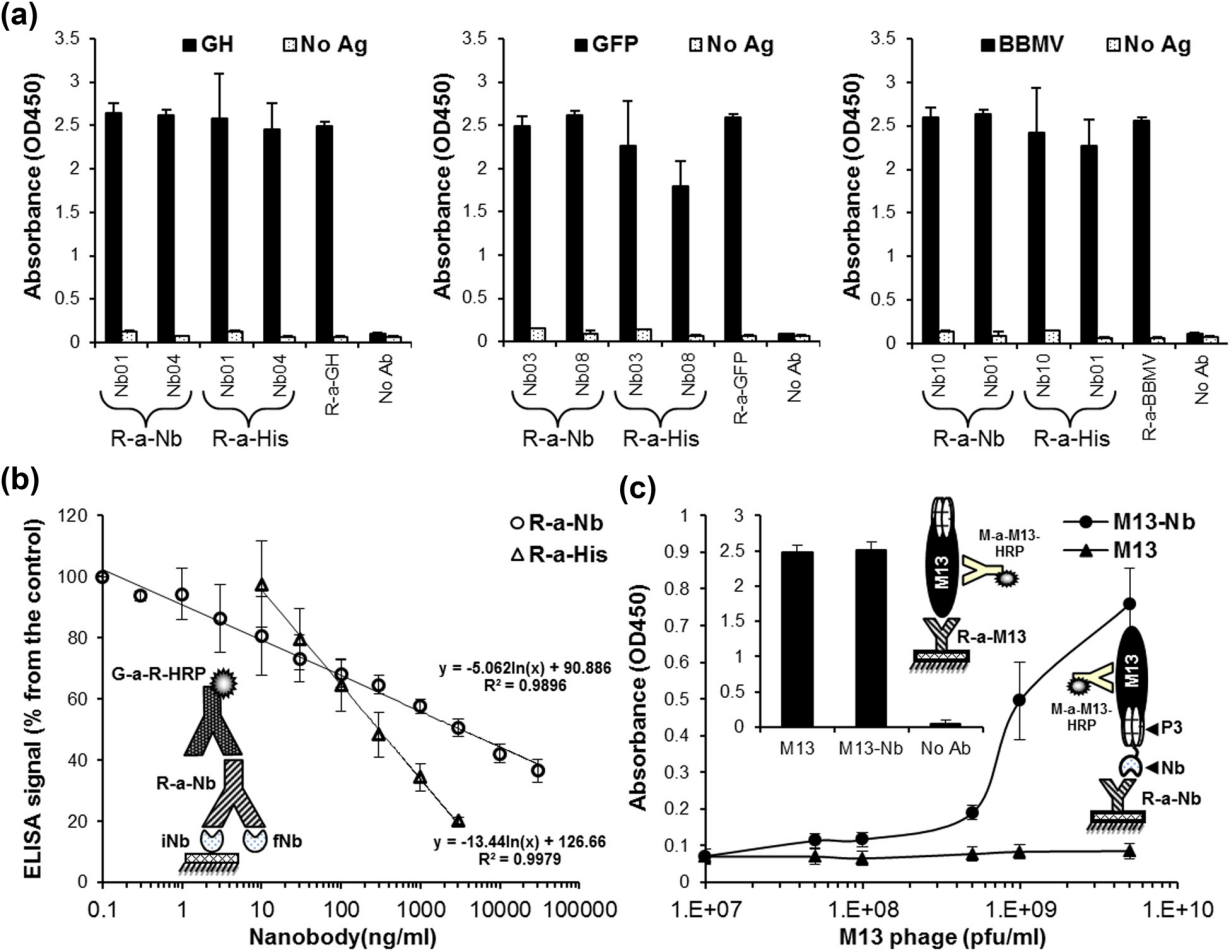
Application of anti-Nb rIgG in Nb immunodetection. (a) Detection of six Nbs (NbGH01/NbGH04, NbGFP03/NbGFP08, and NbBBMV01/NbBBMV10) was done using ELISA in the absence (No Ag) or the presence of immobilized specific antigens (rhGH, GFPuv, and BBMV; 2.5 μg mL^−1^). Detection of antigen-bound Nbs was achieved by either anti-Nb rIgG (1:500) or rabbit anti-6× His antibody (1:2,000). Rabbit anti-hGH, GFP, and BBMV antibodies (1:3,000) were used as controls. (b) An illustration of the principle of the used competitive ELISA is shown, where free (fNb) and immobilized (iNb) Nbs compete to bind to anti-Nb rIgG. Serial concentrations (ng mL^−1^) of free Nbs were incubated with anti-Nb rIgG (R-a-Nb, 1:5,000) or anti-6× His (R-a-His 1:5,000) before transfer to ELISA wells containing immobilized Nbs (1 µg mL^−1^). The linear fit curve of the data set for each condition is represented by the equation [*y* = *a* ln(*x*) + *b*], where *a* is the slope and *b* is the intercept, and the calculated correlation coefficient (*R* ^2^), which is an indicator of the “goodness of fit,” is shown as well. (c) Phage sandwich ELISA was performed to a serial dilution (pfu mL^−1^) of a control (M13) or Nb-displaying phages (M13-Nb) using anti-Nb rIgG (1:500) as a capture immobilized antibody. (c, inset) Both types of phages (M13 or M13-Nb; 10^8^ pfu mL^−1^) were detected by a sandwich ELISA using a rabbit anti-M13 antibody (1:3,000) for capturing. Bound phages were detected by a monoclonal anti-M13-HRP antibody (1:3,000). The principle of each technique is illustrated to the right.

Immunodetection of Nbs using anti-Nb rIgG, compared with anti-6× His antibody, was achieved via indirect ELISA using three different immobilized Nb-specific antigens, hGH, GFP, and BBMV, and their six specific Nbs (two Nbs for each antigen). Bound Nbs to their respective antigens were efficiently detected using either anti-Nb rIgG or anti-6× His antibodies, and the signals were strong and comparable to that given by multiple antigen-specific polyclonal antibodies (Figure 5a). Another interesting application for anti-Nb rIgG was in the quantification of Nbs in mixed samples by a competitive ELISA (Figure 5b). Competitive ELISA is usually used as an analytical method to quantify small molecules in raw samples without a need for a pre-purification step. The principle of Nb-competitive ELISA is that free Nbs in the sample compete with the immobilized ones for binding to a specific antibody. Therefore, we used this system of detection to confirm the sensitivity of anti-Nb rIgG toward Nbs. For this purpose, the optimal effective concentration of anti-Nb rIgG necessary for achieving 75% (EC^75^) of the maximal detection signal of immobilized Nbs, estimated at 1 μg mL^−1^, was used. Different concentrations of Nbs (in ng mL^−1^) were incubated with either anti-Nb rIgG or anti-6× His antibodies and then added into the wells of an Nb-pre-coated microplate and finally revealed by the secondary anti-rabbit antibodies. Absorbance values were inversely proportioned to the number of free Nbs in the samples. In the case of anti-6× His, EC^50^ of detection was 100 ± 10 ng mL^−1^ and the detection range (from EC^99^ to EC^1^) was from 10 ± 0.5 to 3,000 ± 52.5 ng mL^−1^, whereas anti-Nb rIgG showed similar EC^50^ but with a wider detection range from 0.3 ± 0.04 to 30,000 ± 500 ng mL^−1^ (Figure 5b). Similarly, the content of Nbs in bacteria samples could be determined using such a method by comparison with the same standard curve and logarithmic fit equation.

Another interesting domain for testing anti-Nb rIgG was in the phage display technique to detect Nbs exposed to pIII capsid protein of M13 bacteriophage (M13-Nb particles). Applying phage-sandwich-ELISA was possible using immobilized anti-Nb rIgG to capture M13-Nbs before being detected using HRP-conjugated anti-M13 antibody. The detection signal was proportional to the concentration of phage-Nbs in the sample, whereas M13 helper phages (M13), used as a negative control, showed no detection signal (Figure 5c). On the contrary, when an immobilized rabbit anti-M13 antibody was used to capture M13 phages, instead of anti-Nb rIgG, both M13-Nbs and helper M13 resulted in similar detection signals (Figure 5c, inset). In both systems, the principle of the phage-sandwich ELISA was presented by a graphical illustration to the right.

### 3.7 Epitope mapping of the Nb using anti-Nb rIgG

**Figure 6:**
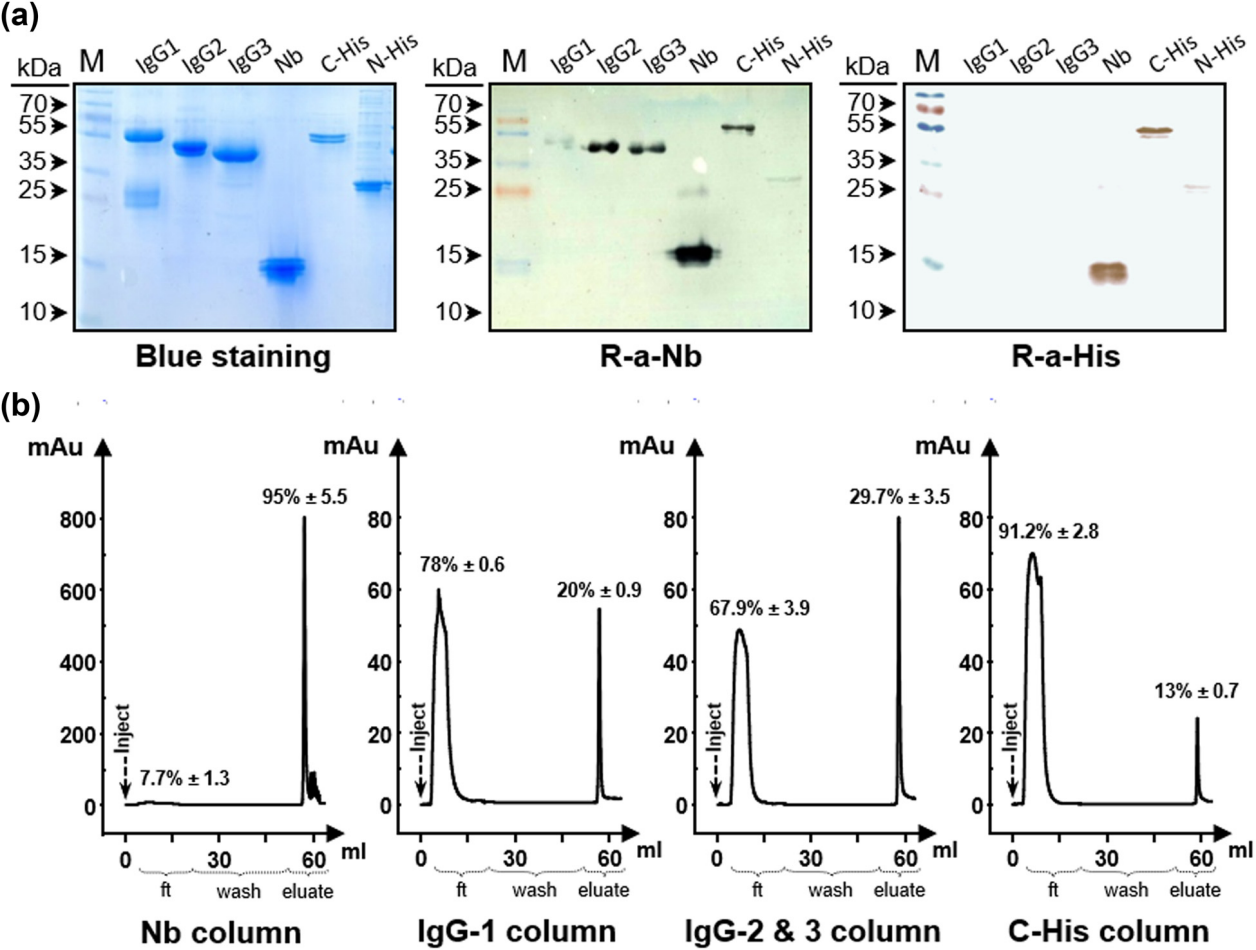
Fractionation of anti-Nb rIgG. (a) SDS-PAGE separation of the different camel subclasses (IgG1, 2, and 3), Nb mixture, C-terminal, and N-terminal 6× His-tagged recombinant proteins (2 μg/lane). Gels were either stained with Coomassie blue or immunoblotted (0.2 μg/lane) with anti-Nb rIgG (1:500) or anti-6× His (1:2,000) antibodies. (b) Five milliliters of anti-Nb rIgG (1 mg mL^−1^) was injected on four different affinity 1 mL columns conjugated to Nb, cIgG1, cIgG2&3, or C-His tagged recombinant protein. Unbound IgGs flow-through (ft) fractions were recovered and then pure fractions were eluted (eluate). The percentages of proteins in the fractions to the total amount injected are shown above the purification peaks.

Then, we evaluated the reactivity of anti-Nb rIgG with the different cIgG subclasses after being prepared from 5 mL of camel blood serum. The cIgG subclasses (IgG1, 2, and 3) were fractionated from the serum sample by differential adsorption on protein-G and protein-A columns (data not shown). The integrity and purity of these fractions were visualized by SDS-PAGE (15%) followed by staining with Coomassie blue (Figure 6a, left panel). Expectedly, cIgG1, as a conventional antibody, showed two distinct bands: one of the heavy chains (∼55 kDa) and a smaller one of the light chains (∼25 kDa), whereas HCAbs (IgG2 and 3) showed smaller single bands related to their heavy chains lacking the CH1 domain. The band of cIgG2 (∼50 kDa) was notably bigger than that of cIgG3 (∼45 kDa) because of the long hinge region characterizing this subclass of antibodies. Nb mixture, loaded in SDS-PAG, appeared as a wide band of ∼15 kDa. Furthermore, two recombinant proteins, with a C-terminal (∼55 kDa) and N-terminal (∼25 kDa) 6× His tags, were loaded on the gel and used as controls for the interaction of anti-Nb rIgG with the recombinant 6× His tag of the Nb. As expected, anti-Nb rIgG was very efficient in detecting Nbs by immune-blotting, and similarly, it detected camel HCAbs (IgG2 and 3) as well as the C-terminal 6× His-tagged protein. The bands that appeared in the case of cIgG1 and N-terminal 6× His-tagged protein were remarkably faint (Figure 6a, middle panel). As a control, the anti-6× His tag antibody was also able to detect Nbs and not any of the other used camel IgGs, and it showed more reactivity toward C-terminal 6× His-tagged protein than to the other recombinant one (Figure 6a, right panel).

**Figure 7:**
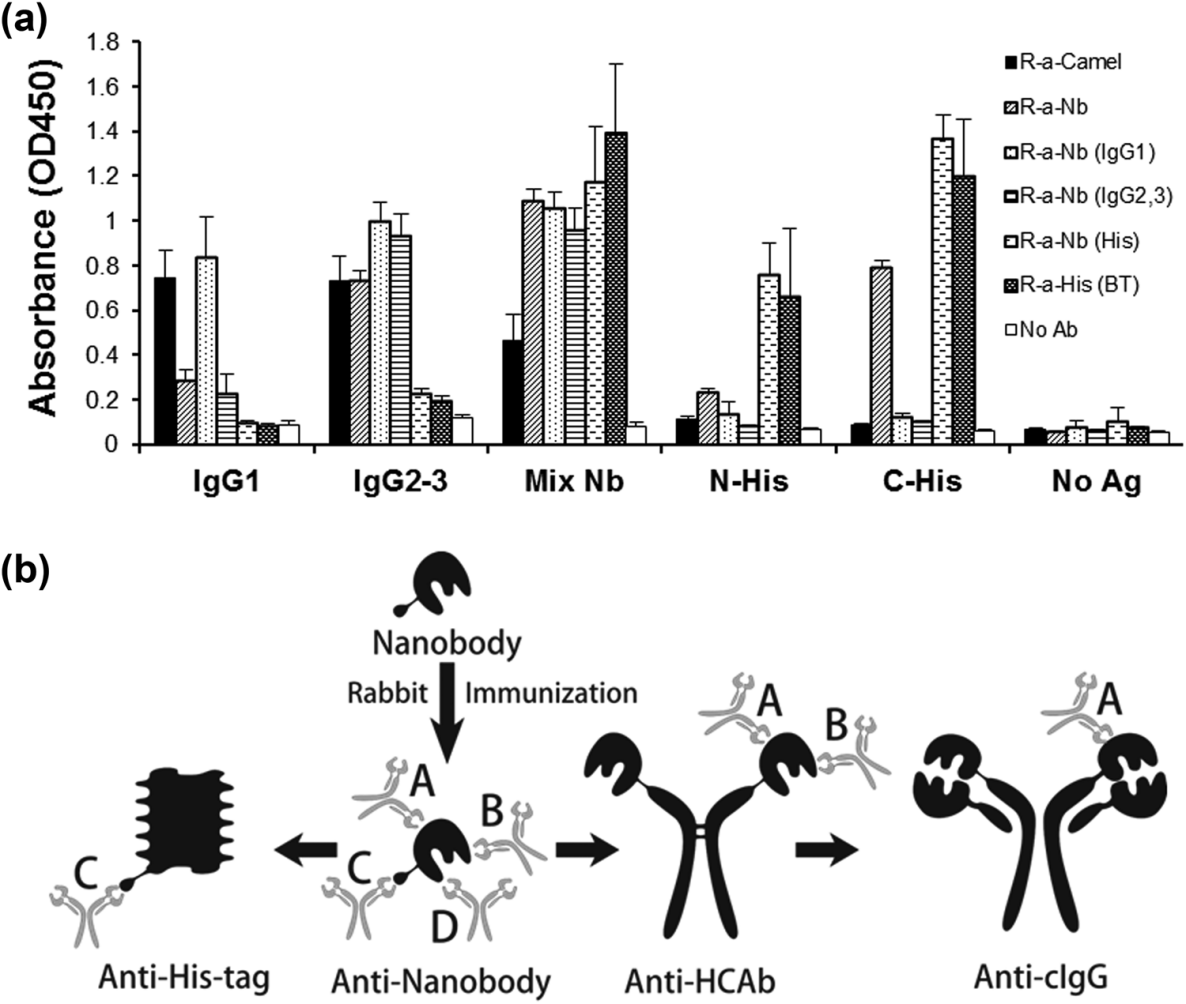
Determination of Nb epitopes recognized by anti-Nb rIgG. (a) Rabbit anti-camel (1:5,000) and anti-6× His (BT) (1:2,000) antibodies as well as the different fractions from the last affinity purification experiment (1 mg mL^−1^, 1:1,000) were tested in ELISA against several antigens: different camel subclasses (IgG1 or IgG2&3), Nb mixture, and N-His- and C-His-tagged recombinant proteins (1 µg mL^−1^). (b) Schematic presentation of the different epitopes of Nb structure that are discriminated by anti-Nb rIgG antibody. Four proposed epitopes are shown: A (anti-cIgG), B (anti-HCAb), C (anti-His), and D (anti-Nb).

The different fractions of anti-Nb rIgG were separated depending on their specificities to shared epitopes between the Nb, cIgG1, HCAb, and 6× His-tagged proteins. These fractions were purified from anti-Nb rIgG by affinity chromatography on three columns after conjugation to cIgG1 (anti-Nb [cIgG1], 17 ± 0.9%), cIgG2&3 (anti-Nb [cIgG2&3], 29.7 ± 3.5%) or the C-terminal 6× His tagged protein (anti-Nb [His], 13 ± 0.7%). Re-purification of the anti-Nb rIgG on the Nb-conjugated column resulted in the recovery of total rIgG in the sample (95 ± 5.5%) (Figure 6b). Testing these different fractions of the anti-Nb rIgG in ELISA using their respective antigens confirmed their sought specificity (Figure 7a). The anti-Nb [cIgG2&3], besides recognizing its specific antigen, was reactive with Nb mixture and not with cIgG1, whereas anti-Nb [cIgG1] recognized all three antigens: IgG1, IgG2&3, and Nb mixture. As expected, anti-Nb [cIgG1 and cIgG2&3] fractions failed in recognizing His-tagged proteins. In contrast, anti-Nb [His] did not recognize any of the camel IgGs but interacted with the Nb mixture, and C-terminal 6× His tagged protein and, to a lesser extent, with the N-terminal one.

The previous experiments suggested that the Nb structure comprises at least four distinct types of epitopes: A, B, C, and D (Figure 7b). Epitopes (types A and B) are associated with the structural domains of Nbs as they form part of camel immunoglobulins. Epitopes (type A) of the Nbs could also be found on the conventional antibodies of camels (cIgG1) and similarly on the HCAbs. Besides the epitopes (type A), HCAbs, and Nbs, as being their derivatives, shared other distinct epitopes (type B) that characterize the heavy-chain antibodies of the camel. The epitopes (type C) are related to the recombinant structure formed by the 6× His tag of the Nb with a great preference for the C-terminal type. Finally, the Nb recombinant structure includes additional epitopes (type D), which are the most abundant (∼37 ± 2.5%) and could be associated with antigen recognition domains that differ between Nbs.

## 4 Discussion

Camelids serum (dromedary, camel, llama, and alpaca) contains naturally occurring HCAbs that are homodimers of heavy chains lacking both the first constant domain (CH1) and complete light chains [5]. It seems that different antigenic sites on the targets are recognizable by the paratopes of conventional antibodies and HCAbs. Therefore, the selection and maintenance of HCAbs in the camelid species were complementary roles in their immune system [56]. The antigen-binding fragment of HCAb is comprised of a single domain, the Nb that can be produced as a soluble recombinant protein in *E. coli*. Nanobodies have a broad range of applications as tools in biotechnology and therapeutic candidates due to their robust nature, small size, ease of production, and high affinity [57]. The bottleneck of applying Nbs, or recombinant antibodies in biotechnology, is related to the unavailability of affordable secondary antibodies as a detection system. Secondary antibodies bind to the antigen-bound primary antibodies to assist in their detection, sorting, and purification. As recombinant proteins, Nbs are mainly detected using expensive antibodies targeting their recombinant tags (6× His, HA, and more). We usually detect immobilized or antigen-bound Nbs with a polyclonal or monoclonal anti-6× His antibody and, to a lesser extent, with an anti-HA antibody when Nbs are displayed on the phages [35,17,46,42]. Nanobodies can be engineered to be tagged with much larger functional domains, such as the crystallized fragment (FC) portion of an antibody when its effector functions and its long half-life are required to be attributed to the Nbs [4], or CH3 when homo- or heterodimers of Nbs are required [58]. Although monoclonal anti-6× His antibodies are very specific, they are very difficult to produce considering the complexities of hybridomas preparation and long steps of affinity purification [59]. Usually, these antibodies target one epitope that is not always in a favorable folding for recognition, especially upon fusion to unpredictable structures like the case of newly retrieved recombinant antibodies. A polyclonal antibody directed to the backbone of Nb could be an interesting detection alternative, resulting in a wide spectrum of detection and better-amplified signal, as several antibody molecules seeking multiple epitopes are involved in the recognition. In this work, we described the production of anti-Nb polyclonal antibodies in the rabbit as a useful tool for detecting Nbs and HCAbs as well as any recombinant proteins with a 6× His tag.

Rabbit polyclonal anti-camel serum is commercially available (Bethyl Laboratories Inc.), and its powerful capacity to equally recognize all camel IgG subclasses and bind their derived Nbs has been proven in this study and a previous study [49]. Past attempts to produce anti-camel monoclonal antibodies in mice resulted in specific ones for IgM [60] and IgG1/IgM [61] but not for the HCAbs (cIgG2 or cIgG3). The explanation for this finding could be that the produced monoclonal antibodies have recognized the light chains that are absent in the HCAbs. Furthermore, the production of several monoclonal antibodies with specificity for the different subclasses of llama IgGs has been described [62]. Besides affinity chromatography of each IgG subclass using its specific antibody, such antibodies were very useful in assessing the participation of llama HCAbs in the immune response against parasite infection. However, only anti-IgG1 mAb could recognize this subclass in all new- and old-world camelids. Besides, anti-IgG2 and anti-IgG3 failed in detecting camel HCAbs. Rabbit anti-camel, anti-Nb, or more specifically, anti-Nb [IgG1] can contribute to monitoring camel immune response as an efficient way to diagnose and control infectious diseases that could affect camel welfare and productivity [63]. Unfortunately, rabbit anti-Nb antibody was only tested against IgGs or Nbs of camel origin due to the unavailability of llama IgGs or Nbs. It is assumed that rabbit anti-Nb antibody should show certain reactivity against llama Nbs due to the high similarity in the amino acid sequences between the Nbs from llama or camel since they belong to the same Camelidae family [64]. Nevertheless, the used plasmids in our experiments, pMES4 or pRMES6, are all derivatives of the pHEN4 plasmid, which is the same plasmid used in the expression of many camel and llama Nbs that all share the presence of similar C-His tag.

In disagreement with a previous study showing that monoclonal reagents prepared for other animal species do not react with camel immunoglobulins [61], total camel IgG was reactive with several commercially available animals antisera. Interestingly, human antiserum, particularly anti-IgG, was very reactive with camel IgG and Nbs, confirming the presumed high homology between llama, camel, and human heavy chains variable domains [64,65,66,67]. This is very important in preparing therapeutic Nbs for human diseases since low immunogenicity could be encountered because of high similarity, and thus, less effort is required for Nb humanization. Furthermore, rabbits immunized against Nbs raised high titer antiserum. Thus, the preparation of anti-Nb HRP as a general conjugated secondary antibody for detecting Nbs in various immunoassays has been performed efficiently [68].

Technically, a genetic procedure is followed to generate Nbs, starting from the immunization of camel, the construction of the library, and finally, a phage display method to select the specific binders in a high throughput way [69]. Throughout this technology, several checkpoints exist to evaluate the correct advancement of the procedure. One important question is the rise of the specific and robust immune response against the injected antigen during camel immunization. The other question routinely asked is to what extent HCAbs are involved in this immune response. The answers to both questions can be provided by a simple ELISA of total camel serum or its purified IgG subclasses used at several dilutions to detect the immobilized antigens. The anti-camel detecting antibody is the key component of such ELISA, which must be reactive toward all camel IgG subclasses. We showed in this work that by fractionation of anti-Nb rIgG through affinity chromatography, several sets of antibodies could be recovered and used in such immune response evaluation. Anti-Nb rIgG was able to detect very low amounts (10 ng mL^−1^) of immobilized Nbs by simple indirect ELISA, and this capacity was enhanced ten times by using competitive indirect ELISA. The last method is important for quantifying Nbs within crude protein samples, such as total bacteria extracts, required in certain applications.

Furthermore, untagging Nbs for humanization purposes and medical administration in humans leaves anti-Nb rIgG as a good choice for the detection and quantitation of these Nbs during manufacturing since it recognizes the main polypeptide backbone and not the extra tags. Furthermore, the final step of the immune methods using anti-Nb rIgG, which requires conjugated secondary antibodies, could be omitted simply by covalent conjugation of this antibody to HRP or alkaline phosphatase enzymes [70]. Interestingly, anti-hGH [42] and anti-GFP [46] are VHH Nbs, but anti-BBMV [17] is VH-like Nb; thus, rabbit anti-Nb has the same ability to detect both types of Nbs, and this is critical in applications that require the detection of untagged Nbs.

Rabbit anti-Nb was previously used to show that the variation in Nb-based sandwich ELISA signals between seven different anti-GFP Nbs is dependent on the affinities of these Nbs rather than the amount used for immobilization [46]. Also, the outstanding performance of our previously described Nbs in detecting their denatured antigens (*sf*GFP fusion proteins [46] or hGH derivatives [42,71]) in immunoblotting could be explained by the use of rabbit anti-Nb for the detection of antigen-bound Nbs rather than using the standard anti-6× His antibody. We used this alternative since our antigens were 6× His tagged as well, but at the N-terminal rather than the C-terminal (the case of Nbs), and the commercial anti-6× His antibody interfered between blotted antigens and their detecting Nbs, but rabbit anti-Nb did not.

A similar exciting finding of the strong reactivity of the rabbit anti-camel (from a commercial source) against Nbs was the focus of our previous work [49]. Despite being a perfect solution for detecting Nbs’ main backbone without the risk of tags (6× His), interference between Nbs and their antigens, and shortage of supply was the major drawback of using this antibody. Our attempts to reestablish similar immunization with cIgG-2 and -3 mixture, rather than with camel total cIgGt, resulted in raising a strong immune response against Nbs in rabbit and not in goat (data not shown). As expected, raised immune response cross-reacted strongly with all camel subclasses in both immunized animals (rabbit and goat) by what was published recently by others [63]. Another interesting finding of rabbit immunization with HCAb was that 1-week interval boosters gave the maximum immune response against Nbs after 21 days rather than 56 days, resulting from 2-week intervals (data not shown). Besides application at different checkpoints along the course of the Nb production procedure, anti-Nb rIgG might have great potential in some special applications of camel IgGs and their Nbs. Isolation and quantification of camel conventional and heavy-chain antibodies in camel milk, as done in a previous study [72], can be achieved in a single step using anti-Nb[IgG1] and anti-Nb[IgG2 & 3].

Among all our immunization attempts, chicken immunization with Nbs was very disappointing. Many recent reports described the fantastic potential of chicken IgY antibody as an alternative for IgG from mammals animals [54]. Besides its distinguishable structure, IgY is very useful when the effector functions related to the FC portion of the antibody are favorably not required for certain applications. Antibody titrations of immunized chickens can reach up to 1:1,000,000, and since chickens can lay eggs almost every day, and the yolk of an immunized hen’s egg contains a high concentration of IgY (more than 50 mg of pure IgY), chickens are considered as an affordable source of antibodies for research and applications [54]. We were hoping that by producing anti-Nb IgY, a great tool for Nbs detection and purifications will be available, especially since the interspace distance between camelids and avian is high enough so Nbs are extremely strange for the chicken immune system. Unfortunately, no previous attempts to immunize chickens with Nbs could be found to compare our results, and the real reason behind our failure in immunizing three different hens with Nbs is inconceivable. Instead, it was shown that immunizing hens with camel IgG resulted in anti-camel IgY capable of recognizing all camel subclasses in immunoblotting with strong cross-reactions with IgGs from other mammal animals without providing any indication about the titer of such IgY [73].

## 5 Conclusions

In summary, a mixture of six different Nbs, expressed using a special T7 plasmid constructed in this work, was prepared and used to produce a specific anti-Nb antibody as a general detection reagent for camel antibodies, conventional cIgG, and HCAb, and the Nbs derived from them. Immunizing with this mixture failed to solicit a significant immune response in chickens, but succeeded in rabbits. Rabbit anti-Nb rIgG was able to detect immobilized or antigen-bound Nbs, and its capacities have been demonstrated in dosing impure Nbs by competitive ELISA in detecting Nbs displayed on the tips of M13 phages by sandwich ELISA, and in revealing denatured Nbs in immunoblotting. As expected and because of shared epitopes, anti-Nb rIgG cross-reacted with cIgG, HCAbs, and a C-terminal 6× His tagged recombinant proteins. Rabbit anti-Nb rIgG, besides its importance as a general detector for camel immunoglobulins and recombinant 6× His tagged proteins, is a promising tool for the checkpoints throughout the recombinant Nb technology.
